# Supplementary material for: Viridicatol Isolated from Deep-Sea Penicillium Griseofulvum Alleviates Anaphylaxis and Repairs the Intestinal Barrier in Mice by Suppressing Mast Cell Activation
Source: Mar Drugs. 2020 Oct 16;18(10):517. doi: 10.3390/md18100517 (PMC7590054; doi:10.3390/md18100517)
Supplement: Supplementary file 1 [file marinedrugs-18-00517-s001.pdf]

Supplementary Methods

HRESIMS spectra were conducted on a Xevo G2 Q-TOF mass spectrometer (Waters Corporation, Milford, MA, USA). NMR spectra were recorded on a Bruker 400 MHz spectrometer (Bruker, Fällanden, Switzerland).

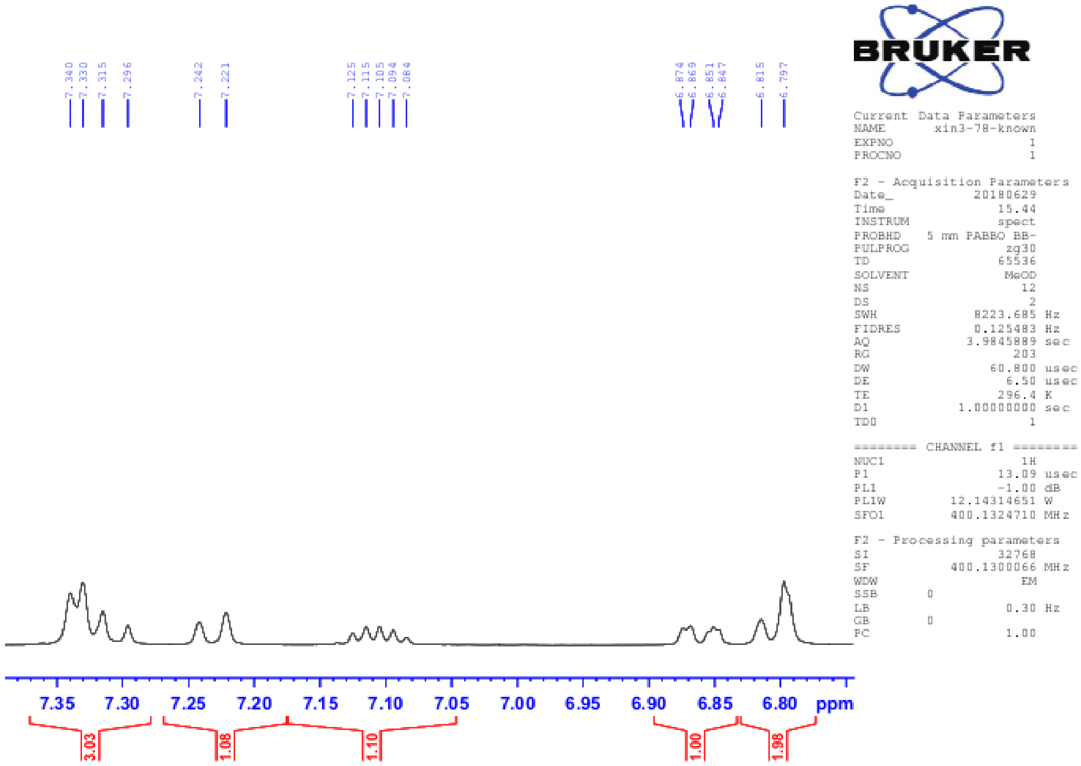

Fig. S1. ^{1}H NMR spectrum of viridicatol in CD_3OD (400 MHz).

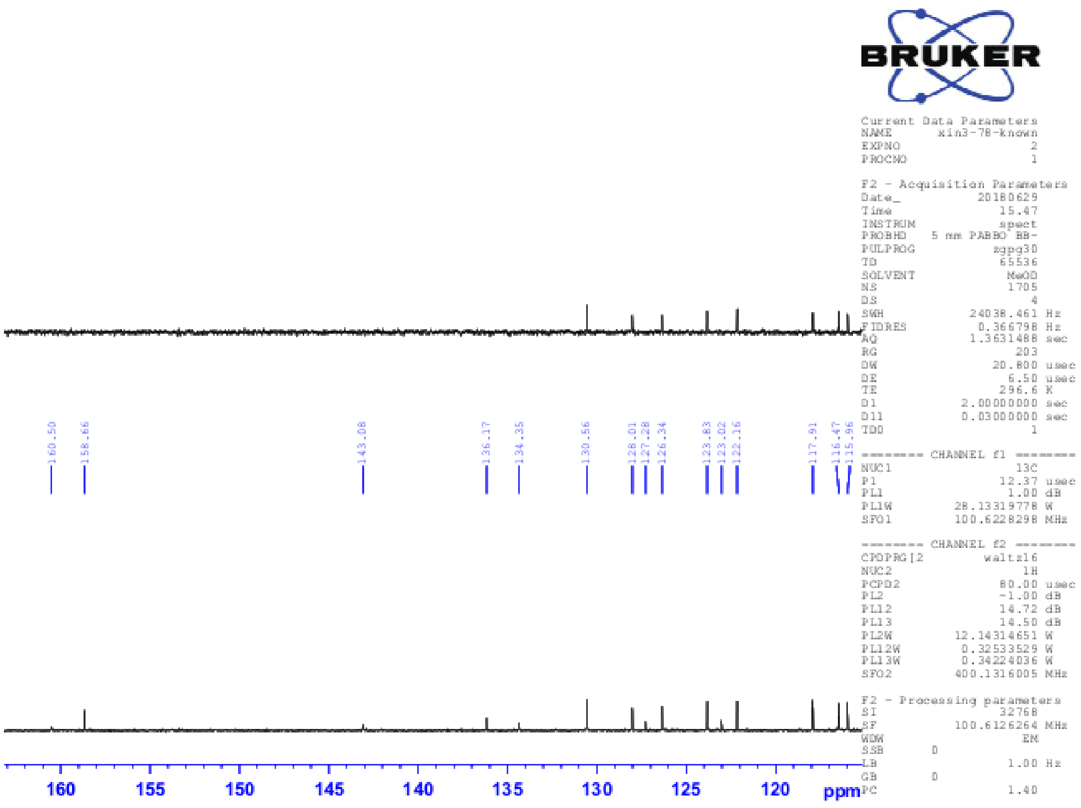

Fig. S2. ^{13}C NMR spectrum of viridicatol in CD_3OD (100 MHz).

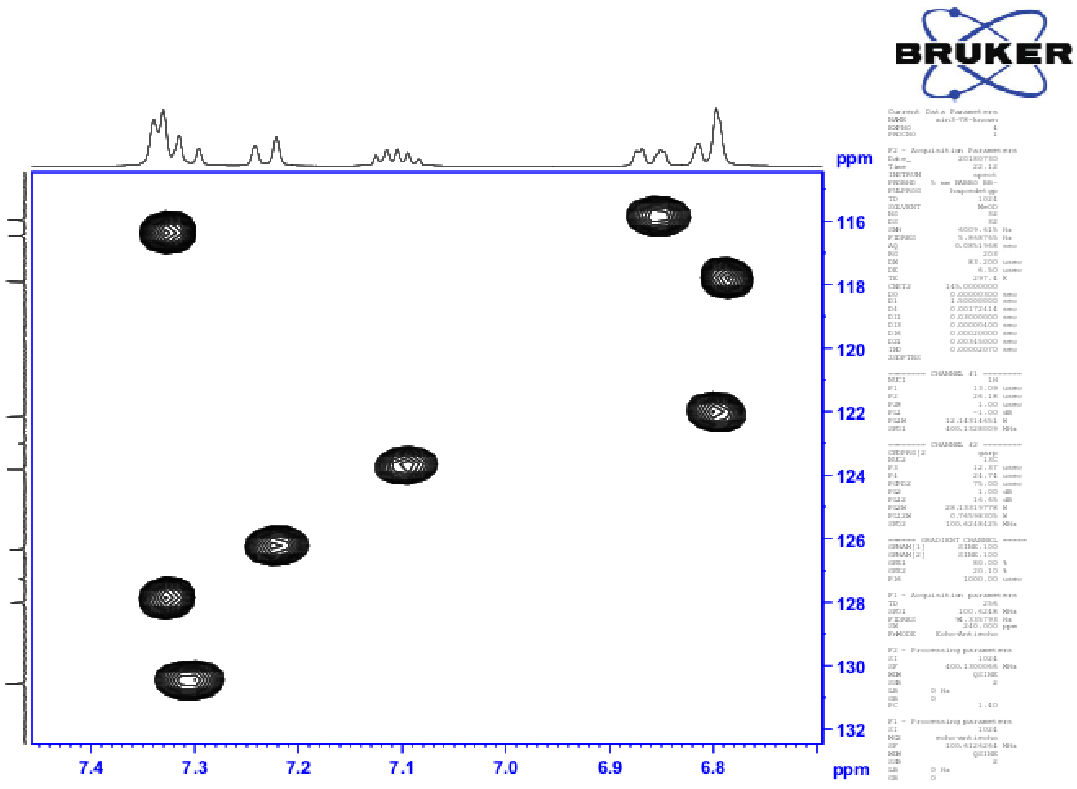

Figure S3. HSQC spectrum of viridicatol in CD_3OD.

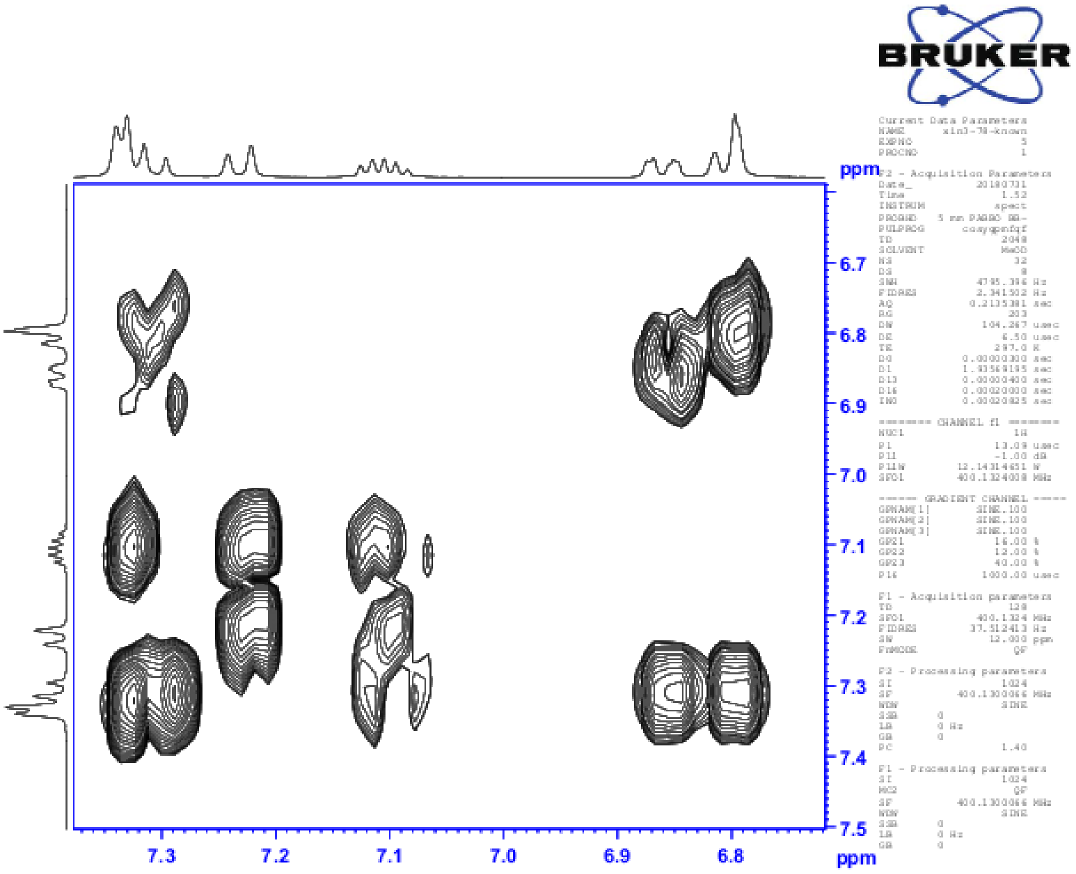

Fig. S4. COSY spectrum of viridicatol in CD_3OD.

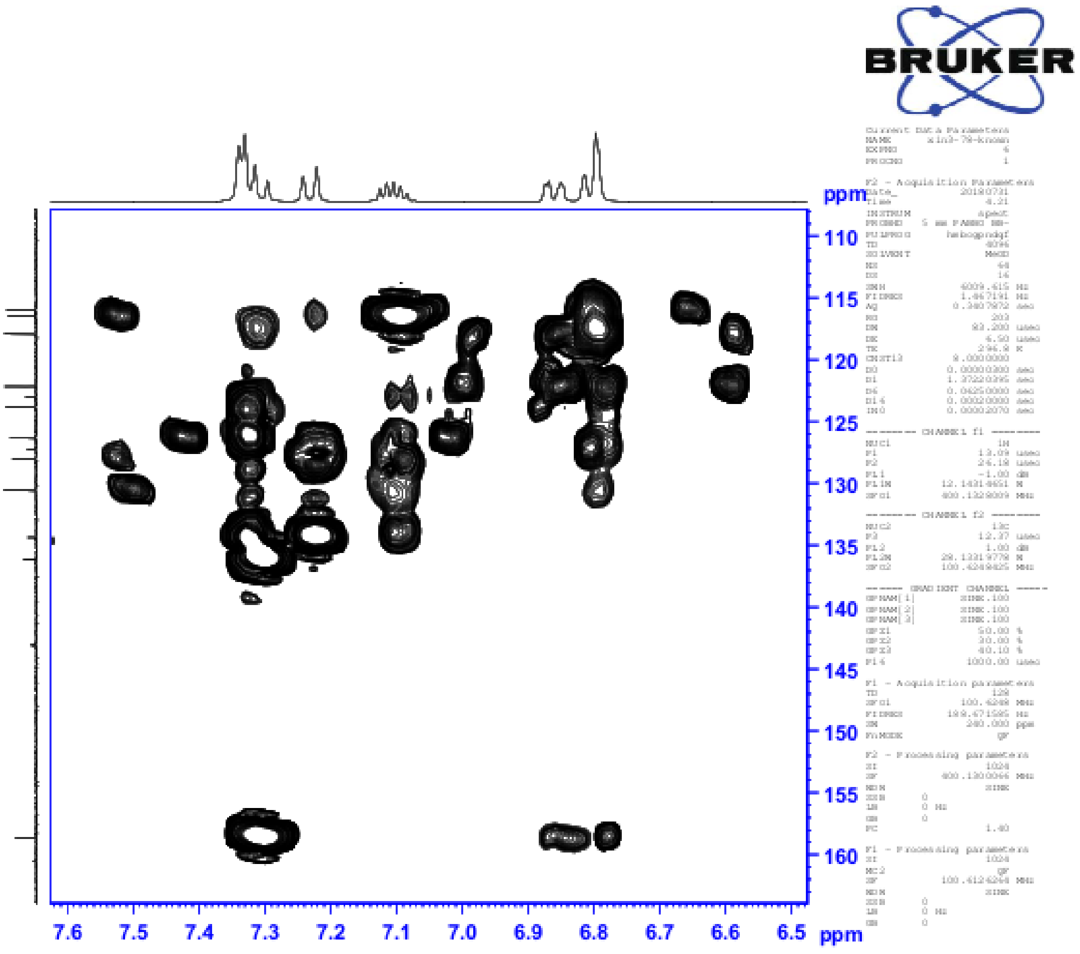

Fig. S5. HMBC spectrum of viridicatol in CD_3OD.

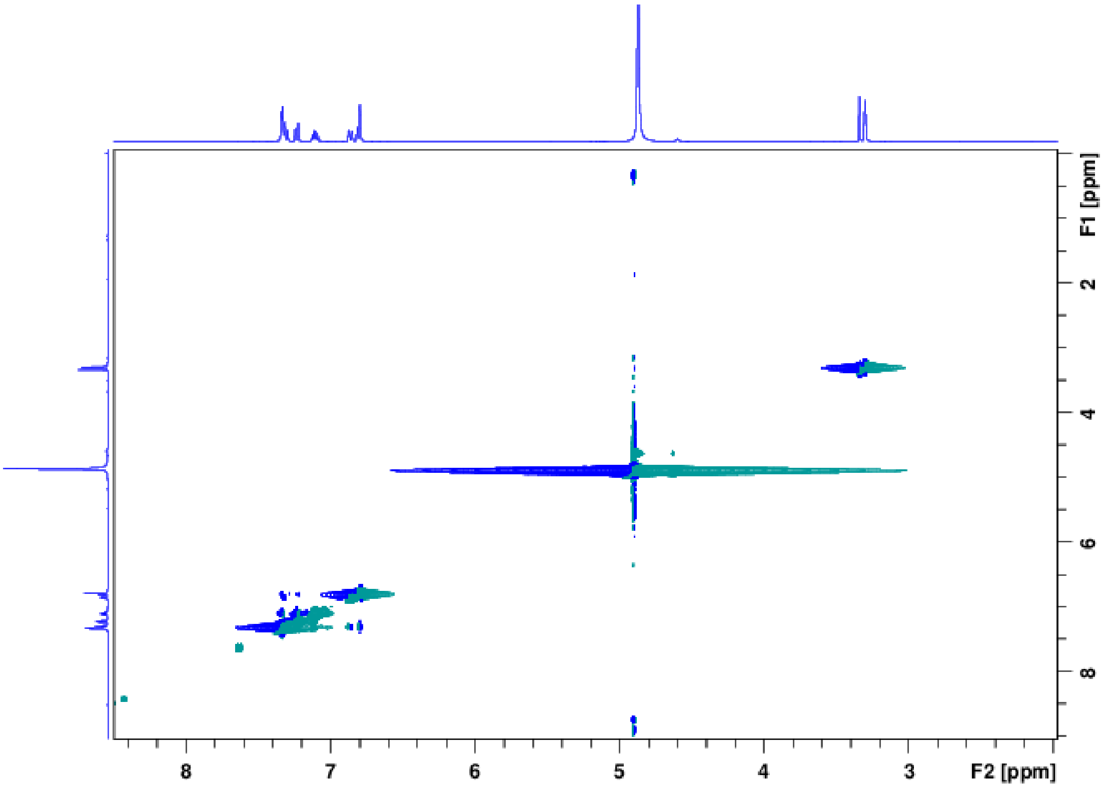

Fig. S6. NOESY spectrum of viridicatol in CD_3OD.

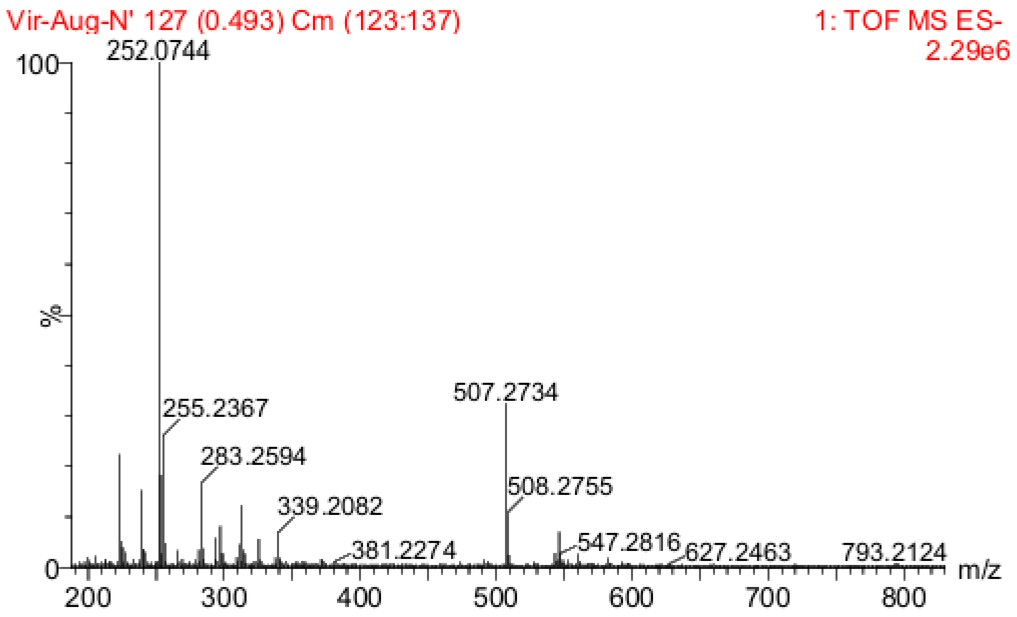

Fig. S7. The HR-ESI-MS spectrum of viridicatol.
